# Apolipoprotein E in Alzheimer’s disease: molecular insights and therapeutic opportunities

**DOI:** 10.1186/s13024-025-00843-y

**Published:** 2025-04-24

**Authors:** Abdel Ali Belaidi, Ashley I. Bush, Scott Ayton

**Affiliations:** 1 The Florey Institute of Neuroscience and Mental Health, Parkville, VIC 3052 Australia; 2 The Florey Department of Neuroscience and Mental Health, University of Melbourne, Parkville, VIC 3052 Australia

**Keywords:** Alzheimer’s disease, Apolipoprotein E, ApoE Christchurch, Apolipoprotein E receptors, APOER2, Neuroinflammation, Autophagy, Neurodegeneration, Lipid peroxidation, Ferroptosis

## Abstract

Apolipoprotein E (*APOE-* gene; apoE- protein) is the strongest genetic modulator of late-onset Alzheimer’s disease (AD), with its three major isoforms conferring risk for disease ε2 < ε3 < ε4. Emerging protective gene variants, such as *APOE* Christchurch and the COLBOS variant of *REELIN*, an alternative target of certain apoE receptors, offer novel insights into resilience against AD. In recent years, the role of apoE has been shown to extend beyond its primary function in lipid transport, influencing multiple biological processes, including amyloid-β (Aβ) aggregation, tau pathology, neuroinflammation, autophagy, cerebrovascular integrity and protection from lipid peroxidation and the resulting ferroptotic cell death. While the detrimental influence of apoE ε4 on these and other processes has been well described, the molecular mechanisms underpinning this disadvantage require further enunciation, particularly to realize therapeutic opportunities related to apoE. This review explores the multifaceted roles of apoE in AD pathogenesis, emphasizing recent discoveries and translational approaches to target apoE-mediated pathways. These findings underscore the potential for apoE-based therapeutic strategies to prevent or mitigate AD in genetically at-risk populations.

## Apolipoprotein E in the brain

Apolipoprotein E (*APOE-* gene; apoE- protein) is a multifunctional protein involved in lipid transport, neuroinflammation, and neuronal repair, playing a crucial role in brain physiology and the pathogenesis of Alzheimer’s disease (AD) [1, 2]. ApoE is a secreted, lipid-transporting protein in both the periphery and the central nervous system (CNS) with plasma concentrations of ≈55 µg/ml and about 10-fold lower concentrations in CSF (6.5–7.3 µg/ml) [3]. ApoE is primarily synthesized by the liver and brain, where it facilitates the transport and redistribution of lipids. In the CNS, apoE is primarily secreted by astrocytes and, to a lesser extent, by microglia and neurons under certain pathological conditions [4].

### Structure and isoforms

**Fig. 1:**
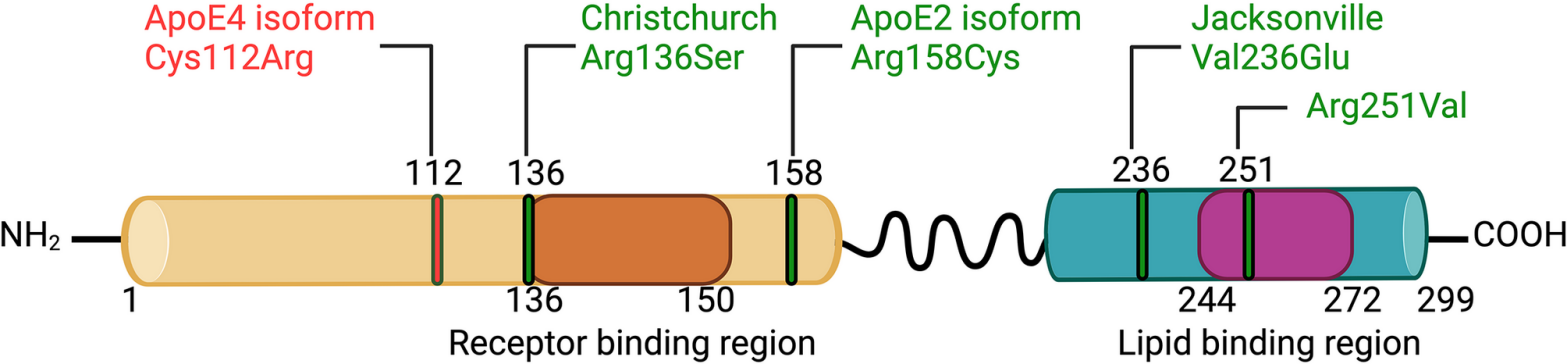
ApoE domain structure and associated variants. Domain organization of the human apoE protein is highlighted including amino acid changes associated with the AD risk isoform apoE4, the protective isoform apoE2 and 3 additional protective variants: the Christchurch variant, the Jacksonville variant and the Arg251Val variant

ApoE is a 299-amino acid glycoprotein characterized by two distinct functional domains: an N-terminal receptor-binding domain (amino acid residues 136–150) and a C-terminal lipid-binding domain (residues 244–272) (Fig. 1). The APOE gene, located on chromosome 19, encodes three major isoforms: apoE2, apoE3, and apoE4, differing by single amino acid substitutions at positions 112 and 158. Specifically, apoE2 contains cysteine at both positions (Cys112, Cys158), apoE3 contains cysteine and arginine (Cys112, Arg158), and apoE4 contains arginine at both positions (Arg112, Arg158).

These single amino acid substitutions lead to significant structural differences among the isoforms, profoundly influencing their function. ApoE2 exhibits a notable conformational alteration in its N-terminal receptor-binding domain compared to apoE3, markedly reducing its affinity for apoE receptors [5, 6]. Consequently, this isoform demonstrates decreased receptor-mediated clearance, resulting in elevated plasma levels. Clinically, apoE2 homozygosity is associated with a rare disorder called type III hyperlipoproteinemia, characterized by impaired lipid metabolism [7].

ApoE4’s structural alteration involves disruption of salt bridges and the formation of abnormal domain interactions within its C-terminal domain [8]. Additionally, apoE4 exhibits a propensity to form an unstable molten globule intermediate state, accelerating its degradation [9]. These structural and stability issues significantly impair apoE4’s lipid transport efficacy and receptor interaction capacity.

These isoform-specific structural differences have important functional consequences, especially regarding lipid transport, receptor binding, and protein stability. Plasma apoE levels correlate with isoform stability and receptor-binding properties (apoE2 > apoE3 > apoE4), reflecting the decreased stability of apoE4 and the reduced receptor-mediated clearance of apoE2 [10–12]. Humanized apoE mouse models (constitutive *knock in* models) exhibit similar correlations between *APOE* genotype and brain apoE levels [10–12]. It should be noted that while in vitro studies and humanized apoE mouse models have provided valuable insights into isoform-specific effects of apoE, these models do not fully replicate the complexity of human AD pathology. Key aspects such as the gradual progression of neurodegeneration, the full spectrum of tau pathology are often absent or only partially modeled. Moreover, while many studies in mouse models and cell cultures report *APOE* genotype-dependent differences in protein levels, particularly the trend of apoE2 > apoE3 > apoE4 in both stability and expression, this pattern is not consistently observed in human cerebrospinal fluid (CSF). Human cohort studies often fail to replicate the clear genotype-dependent differences in CSF apoE levels seen in brain tissue or animal models, possibly due to methodological differences, postmortem changes, or tissue compartmentalization [3, 13].

In terms of allele frequency and Alzheimer’s disease (AD) risk, *APOE* ε3 is the most prevalent allele and is considered neutral concerning AD susceptibility [14]. *APOE* ε2, less common, is protective, significantly reducing the risk of AD development [14]. *APOE* ε4 is the strongest genetic risk factor for late-onset AD, considerably increasing the odds of disease onset [14]. A recent large-scale study involving 28,864 participants confirmed this trend, reporting increased AD risk associated with *APOE* ε4 carriage and markedly decreased risk among *APOE* ε2 carriers, especially in *APOE* ε2 homozygotes [15].

### Apolipoprotein E receptors

**Fig. 2:**
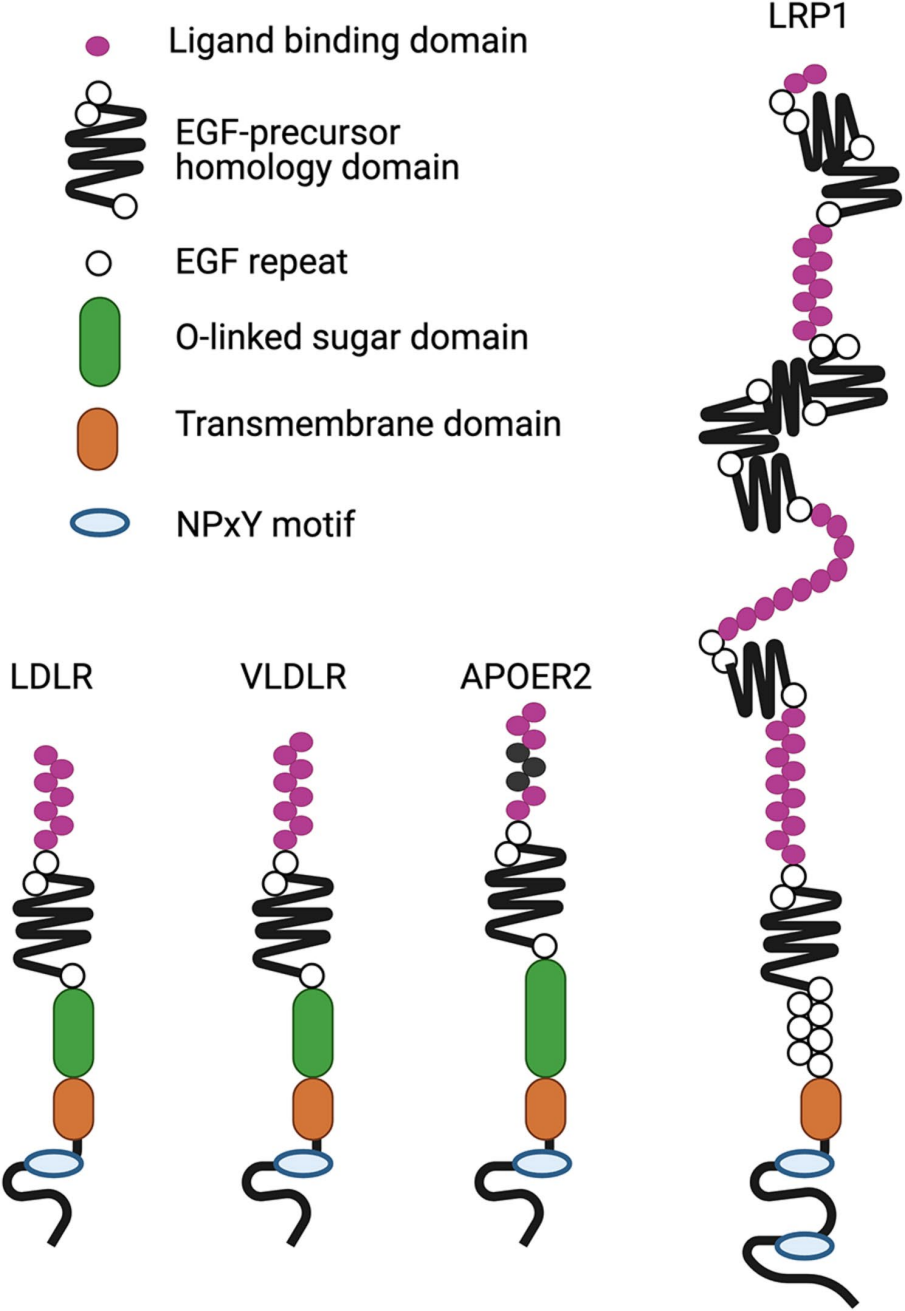
Structural organization of apoE receptors. LDLR, VLDLR, APOER2 and LRP1 share similar structure that includes an extracellular ligand binding domain, EGF-precursor homology domain, an O-linked sugar domain, a transmembrane domain, and an intracellular cytoplasmic domain that contains an NPxY motif

ApoE exerts its physiological effects in the brain by binding to a family of receptors, collectively known as the low-density lipoprotein (LDL) receptor family. These receptors are involved in endocytosis of extracellular ligands and share a similar basic structure consisting of an N-terminal ligand binding domain with varying numbers of ligand-binding repeats, an epidermal growth factor (EGF) precursor homology composed of EGF-repeats, an O-linked sugar domain, a transmembrane domain and a cytoplasmic domain containing varying numbers of NPxY motifs (Fig. 2). The most common members of the LDL receptor family that are expressed in the CNS in various cell types, including neurons, astrocytes, and endothelial cells are low-density lipoprotein receptor (LDLR), low-density lipoprotein receptor-related protein 1 (LRP1), very-low-density lipoprotein receptor (VLDLR), and apolipoprotein E receptor 2 (ApoER2; also known as LRP8).

LDLR is the primary receptor involved in lipid uptake and endocytosis and its role in cholesterol homeostasis is well understood [16]. LDLR preferentially binds apoE3 and apoE4 isoforms with high affinity, whereas apoE2 shows substantially reduced affinity due to structural changes in its N-terminal domain [5, 6]. This difference in LDLR binding was highlighted in a recent study that used surface plasmon resonance to measure binding affinities of lipidated apoE isoforms to LDLR extracellular domain [17]. ApoE3 and apoE4 demonstrated similar high binding affinities (16 and 9 nM, respectively), whereas apoE2 exhibited a weaker binding affinity of approximately 800 nM [17]. Interestingly the study showed that the protective apoE Christchurch variant exhibited also weaker binding affinity to LDLR similar to apoE2 [17]. LRP1 plays also a key role in lipid uptake but acts additionally as a scavenger for a diverse spectrum of ligands including amyloid beta (Aβ) and tau, which are components of the pathological hallmarks of AD [18–20]. ApoE isoforms exhibit differential binding affinities for LRP1, with apoE3 generally demonstrating optimal receptor interactions compared to apoE2 and apoE4; the impaired binding of apoE4 to LRP1 can lead to reduced clearance efficiency of Aβ, enhancing AD pathology [21, 22]. Expression patterns differ between LDLR and LRP1, with LDLR highly expressed on glial cells and minimally on neurons, whereas LRP1 expression predominates in neurons [23]. Deletion of LDLR and LRP1 in mice increases brain apoE levels [24, 25]. Conversely, overexpression of LDLR in the brain decreases apoE levels [26].

Unlike LDLR and LRP1, VLDLR and apoER2 are well known for their role in the development of the central nervous system via binding to reelin and activation of the reelin signaling pathway [27, 28]. The reelin signaling pathway is a critical regulator of brain development and synaptic function [29]. Reelin, a large extracellular matrix glycoprotein, binds to apoER2 and VLDLR, triggering binding of the intracellular adaptor protein Disabled-1 (Dab1) at the NPxY motif present on the cytoplasmic loop, which leads to Dab1 phosphorylation and activation of tyrosine kinases [29]. This activation initiates a downstream signaling cascade that influences neuronal migration, dendritic growth, and synaptic plasticity [29]. Deletion of VLDLR and apoER2 in mice leads to disruption of the cortical layers in hippocampus and cerebellum that mimics the phenotype observed in reelin or mDab1 knockout mice [27]. Aside from the overlapping functions of VLDLR and apoER2, both receptors have also distinct properties that separate them [28]. In particular, apoER2 plays an important role in regulating intracellular selenium levels by binding selenoprotein P, which is the major selenium transport protein of the body and mediates its endocytosis and cytoplasmic selenium release [30]. This apoER2 function is important in indirectly protecting cells from lipid peroxidation and ferroptosis (discussed in Sect. “Autophagy”).

ApoE binds to another important class of receptors called heparan sulfate proteoglycans (HSPGs), which are widely expressed in glia and neurons. HSPGs are complex molecules found on the cell surface and in the extracellular matrix, composed of a core protein and heparan sulfate glycosaminoglycan chains. In the context of apoE function, HSPGs act as co-receptors or facilitators for apoE-receptor complexes, aiding in the sequestration of apoE particles and subsequent binding to LRP1 [31]. Recent studies implicate apoE-HSPG interaction in tau pathogenesis in AD, following the discovery of the protective apoE Christchurch variant, which displays weaker binding affinity to HSPGs [32]. This reduced affinity correlates with diminished tau propagation between neurons, highlighting a potential therapeutic target [33] (discussed in Sect. “Apolipoprotein E Christchurch variant”).

## Apolipoprotein E biology

**Fig. 3:**
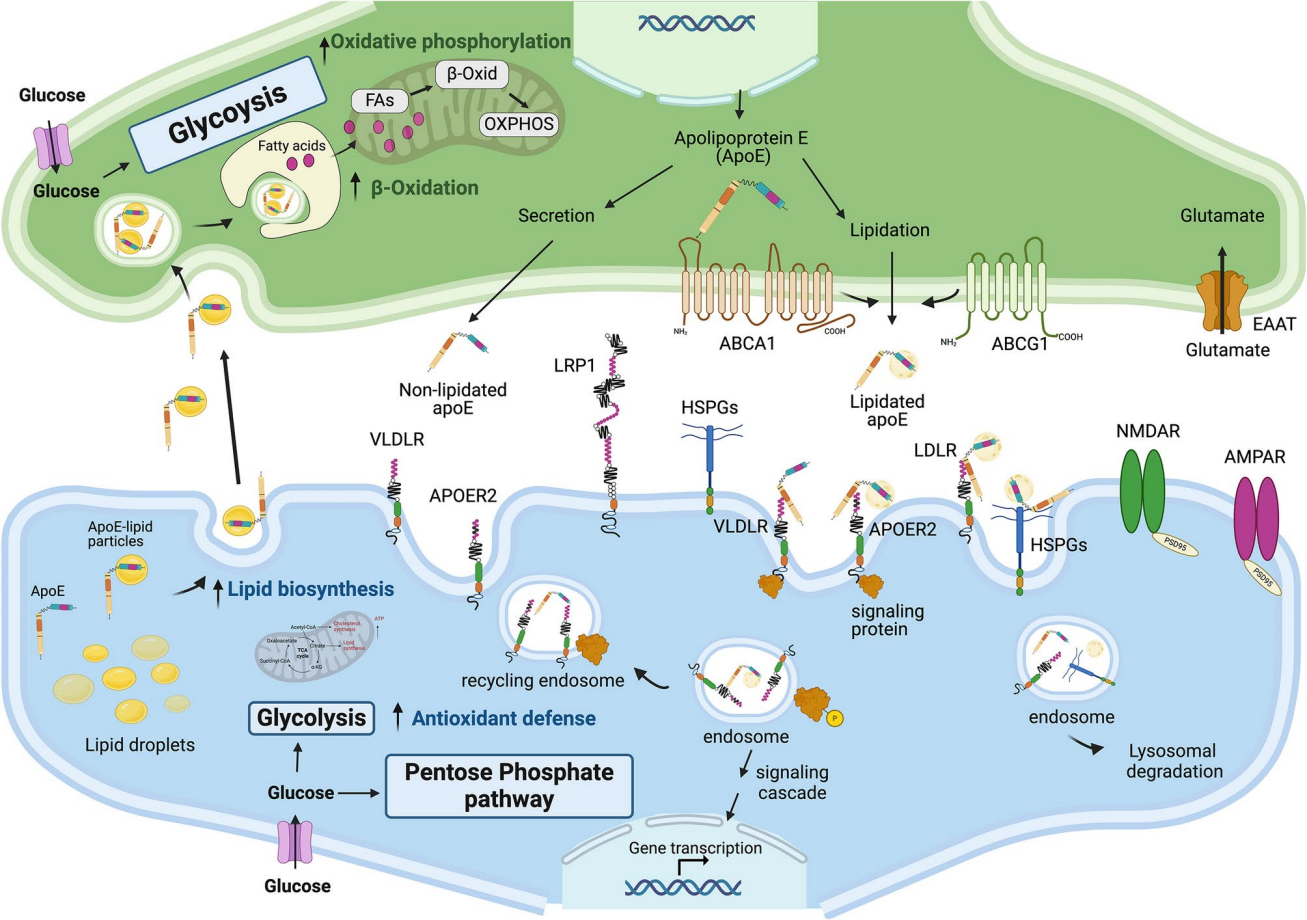
ApoE biology in the brain. ApoE is mainly expressed by astrocytes in the brain. Upon expression, apoE is lipidated mainly by the ABCA1 transporter, which is located at the plasma membrane of astrocytes and ABCG1, which is present on the plasma membrane of astrocytes and neurons. Secreted apoE binds to different receptors expressed on neurons (and other cells) that are involved in endocytosis (LDLR, LRP1, HSPG) or signaling (apoER2, VLDLR) and affects endosomal trafficking and receptor recycling to the plasma membrane. ApoE is also required for the transport of excess oxidized fatty acids that are generated during neuronal activity to astrocytes, which are either stored in lipid droplets or used for energy production via β-oxidation

The brain is the most cholesterol-rich organ with 30% of the body’s cholesterol, where it plays an essential role in the maintenance of neuronal functions. Peripheral cholesterol cannot cross the blood-brain-barrier and brain cholesterol is entirely synthesized locally mostly by glial cells [34]. Cholesterol transport is mediated by apolipoproteins that associate with phospholipids and other lipids to form HDL-like particles. The major apolipoproteins in the CSF are apoE and apoA-I with lower levels of apoA-II, apoJ and apoD [34, 35]. While brain apoE is entirely synthesized in astrocytes, apoA-I is transferred from plasma to the CNS and is not synthesized in the brain [34]. Upon expression, apoE lipidation in the CNS is mediated by the action of lipid transporters from the ATP binding cassette (ABC) transporter family such as ABCA1 and ABCG [36]. ABCA1 in astrocytes mediates cholesterol and phospholipid efflux onto newly expressed apoE leading to the formation of initial discoidal-apoE particles. During maturation, further cholesterol and phospholipids are incorporated into the apoE particles by ABCA1 in astrocytes and the action of ABCG1, which is expressed in both astrocytes and neurons [36]. ABCA1 and ABCG1 expression is tightly regulated in astrocytes by the liver X receptors (LXRs) and the retinoid X receptor (RXR) that belong to the type II family of nuclear receptors. LXR and RXR form heterodimers that respond to changes in intracellular cholesterol by modulating the transcription of their target genes including ABCA1, ABCG1 and apoE (Fig. 3) [37].

The apoE role in lipid transport is critical for maintaining neuronal membrane integrity, and supporting essential functions such as axonal growth, neurite formation, vesicular transport, synaptic plasticity, and repair processes. ApoE participates in multiple biological processes and metabolic pathways essential for maintaining cellular homeostasis in the CNS beyond its well-known role in lipid transport [38, 39]. ApoE’s functions in lipid, glucose, and iron metabolism, as well as in cellular processes like autophagy and ferroptosis, underscore its complexity and relevance in neurodegenerative diseases, particularly AD. ApoE has been also linked with cellular senescence in cell culture and human postmortem studies with effect related to cholesterol and lysosomal pathway [40, 41]. This section explores some of the diverse roles of apoE across these pathways that intersect with its role in AD.

### Lipid metabolism

ApoE mediates lipid transport through interactions with LDLR and LRP1 and subsequent receptor-mediated endocytosis into cells, where the lipids are released and used to support multiple processes such as membrane extension and synaptogenesis [42, 43]. The structural differences between the apoE isoforms impact on lipid binding/transport and associated changes, which result in an isoform specific pattern. Apart from impaired lipid transport associated with apoE2 due to impaired receptor binding, multiple studies in cells, mice and patient cohorts reported that apoE4 is less lipidated, contains less cholesterol and is associated with smaller lipid complexes compared to apoE3 and apoE2 [44–48]. Furthermore, lipidation has been shown to protect apoE from aggregation and an apoE isoform-dependent aggregation pattern was identified with apoE4 > apoE3 > apoE2 without or in complex with Aβ [49, 50]. Co-culture studies of astrocytes and neurons further showed that apoE4 was less efficient in lipid clearance from neurons [51–53].

It should be also noted that peripheral apoE, which predominantly interacts with triglyceride-rich lipoproteins and LDL, exhibits binding properties that differ from CNS-derived apoE, which is primarily associated with HDL-like particles and has a distinct lipidation profile. Recent lipidomic studies further highlight significant *APOE* allele-dependent alterations in the brains of AD patients [54]. Specifically, analyses of postmortem brain samples identified pronounced shifts in the lipid composition of AD brains carrying different *APOE* alleles. *APOE* ε4 carriers exhibit altered phospholipid profiles, changes in cholesterol distribution, and disturbances in sphingolipid metabolism compared to *APOE* ε3 and *APOE* ε2 carriers [55]. These lipidomic alterations potentially underpin neuronal vulnerability and neurodegenerative processes associated with AD. Notably, differences in phospholipid profiles between *APOE* ε3 and *APOE* ε4 brain samples directly correlate with distinct microglial responses to amyloid pathology; specifically, apoE3-containing lipoproteins promote faster microglial migration and enhanced Aβ uptake compared to apoE4-containing lipoproteins [56]. Collectively, the apoE4 variant appears to be associated with low cholesterol and poor lipidation, which, together with domain interactions, decreases its stability leading to impaired neuronal lipid metabolism and changes in lipidome profile that alters the brain innate immunity response to AD pathologies.

### Glucose metabolism

Glucose is the primary source of energy of the brain, which is mainly taken up by astrocytes due to their tight association with the vasculature. Astrocytes and neurons collaborate metabolically via a proposed astrocyte-neuron lactate shuttle: astrocytes metabolize glucose primarily through glycolysis, producing lactate, which is then transferred to neurons. Neurons convert lactate to pyruvate to fuel ATP production via the citric acid (TCA) cycle, supporting neuronal energy demands [57, 58].

Glucose hypometabolism, which is measured in AD patients by 18-fluorodeoxyglucose positron emission tomography (FDG-PET) is a well-established clinical observation, which associates with disease severity [59–61]. Decreased FDG has been observed in asymptomatic ε4 carriers, and attributed to multiple factors including mitochondrial dysfunction, neuronal death and amyloid and tau pathologies [62, 63]. A direct effect of apoE on glucose metabolism was evidenced by studies of humanized *APOE knock-in* mice, which revealed a link between *APOE* genotype and insulin/insulin-like growth factor (Igf) signaling [64]. Accordingly, apoE2 brain exhibited the highest glucose uptake rate indicated by high levels of the proteins Igf1, insulin receptor substrates (Irs) and glucose transporter 4 (Glut4), that is consistent with the protection conferred by the apoE2 isoform [64]. Further experiments using immortalized astrocytes derived from the *APOE knock-in* mice confirmed a decrease in glucose uptake according to *APOE* genotype with ε2 > ε3 > ε4 [65]. Moreover, *APOE* genotype impacted directly on the metabolic route of glucose through glycolysis, PPP and the TCA cycle, with apoE4 associated with an increase in glucose flux through routes of lactate synthesis, PPP flux and *de novo* nucleotide biosynthesis [65]. ApoE’s impact on glucose metabolism highlights its broader role in maintaining cellular energy balance, particularly in high-energy-demand tissues like the brain and the effect of its apoE4 isoform may contribute to energy deficits and increased oxidative stress, exacerbating neuronal vulnerability and promoting AD pathology.

### Autophagy

Autophagy, a cellular process that removes damaged proteins and organelles, is essential for maintaining cellular health and preventing the accumulation of toxic aggregates. Evidence linking apoE with autophagy was provided by experiments using apoE-expressing astrocytes, which revealed a decreased autophagic flux in apoE4-expressing astrocytes as compared to apoE3 [66]. In addition, autophagy induction by rapamycin enhances Aβ degradation by apoE4-expressing astrocytes, whereas autophagy inhibition by chloroquine blocks Aβ degradation by apoE3-expressing astrocytes [66]. ApoE was also shown to inhibit autophagy in neurons via binding to apoE receptors and activation of the PI3K/AKT pathway resulting in inhibition of the autophagic degradation of ferritin [67]. Recent clinical data indicated a possible mechanism for impaired autophagy in ε4 carriers mediated by repression of FOxO3a, which is a transcription factor that regulates various autophagy-related genes [68]. Accordingly, decreased FOxO3a expression in ε4 carriers resulted in lowered gene products including atg12, Beclin-1, BNIP3 and PINK1, leading to dysfunction of both autophagy and mitophagy in *APOE* ε4 carriers, correlating with prominent tau accumulation [68]. Another line of evidence linking apoE4 with impaired autophagy was provided by another study that showed that apoE4 binds to the Coordinated Lysosomal Expression And Regulation (CLEAR) DNA motif and mediates the repression of the transcription factor EB (TFEB) that plays a crucial role in regulating various genes required for autophagy and lysosome biogenesis [69]. These finding suggest that apoE4 is associated with impaired autophagy that adversely impacts the capacity of astrocytes to clear protein aggregates such as amyloid and tau aggregates. Enhancing autophagy has emerged as a therapeutic strategy in AD, and understanding how apoE modulates this process could inform new approaches to support cellular clearance mechanisms.

### Iron metabolism

Iron elevation in the brain is a well-recognized biochemical change associated with multiple neurodegenerative diseases including AD and has been reported in multiple studies using postmortem analyses or imaging techniques [70–75]. Abnormal accumulation of iron in brain tissue has the potential to cause damage by oxidative stress [70, 76, 77]. However, a recent clinical trial using the iron chelator deferiprone, which can cross the blood-brain-barrier (BBB) and lower brain iron, induced worsening of cognitive performance in people with amyloid-confirmed mild cognitive impairment (MCI) and early AD [78]. This highlights the essential role of brain iron for cognitive function, and the complex relationship between iron and disease progression in AD. Indeed, an unusual relationship between iron, *APOE* genotype and cognitive decline was reported in MCI and AD subjects from the Alzheimer Disease Neuroimaging Initiative (ADNI) cohort. CSF ferritin (the major iron storage protein) predicted marked acceleration of cognitive decline over 7 years in pre-symptomatic ε4 carriers, while no correlation was observed in non-carriers [79, 80]. A similar association was also reported using data from the Swedish BioFINDER cohort and the Rush Memory and Aging Project (MAP) cohort, which showed that elevated CSF ferritin predicted AD in ε4 carriers [67, 81]. Interestingly, the study showed that *APOE* ε4-associated change in iron (when directly measured in post-mortem cases) is dependent on disease stage, and therefore the effect may not be a direct influence of the apoE protein on brain iron, rather, *APOE* genotype changes the risk attributable to iron [67]. People with *APOE* ε4 are at much greater risk of developing AD and the study found that those ε4 subjects who had comparatively low iron had an equivalent risk of AD compared to those without ε4, but this risk rapidly increases as a function of iron, which is consistent with prior reports [67, 80–82].

A physiological relationship between apoE and iron was further evidenced by mice studies that showed that apoE deficiency induces an age-dependent accumulation of iron in liver and spleen due to an imbalance between iron absorption via transferrin receptor 1 (TfR1) and export via ferroportin 1 (Fpn1) [83]. A follow up study demonstrated that apoE deficiency induces iron elevation in the brain of mice in an age-dependent manner and correlates with elevated markers of neuroinflammation and oxidative stress [84]. These results demonstrates that apoE is required to regulate iron levels in the brain and that changes in apoE levels, as observed in AD patients, may result in iron dysregulation, oxidative stress and neuronal injury. This is supported by direct evidence correlating decreasing CSF apoE levels with cognitive decline and risk for AD [85].

### Ferroptosis

**Fig. 4:**
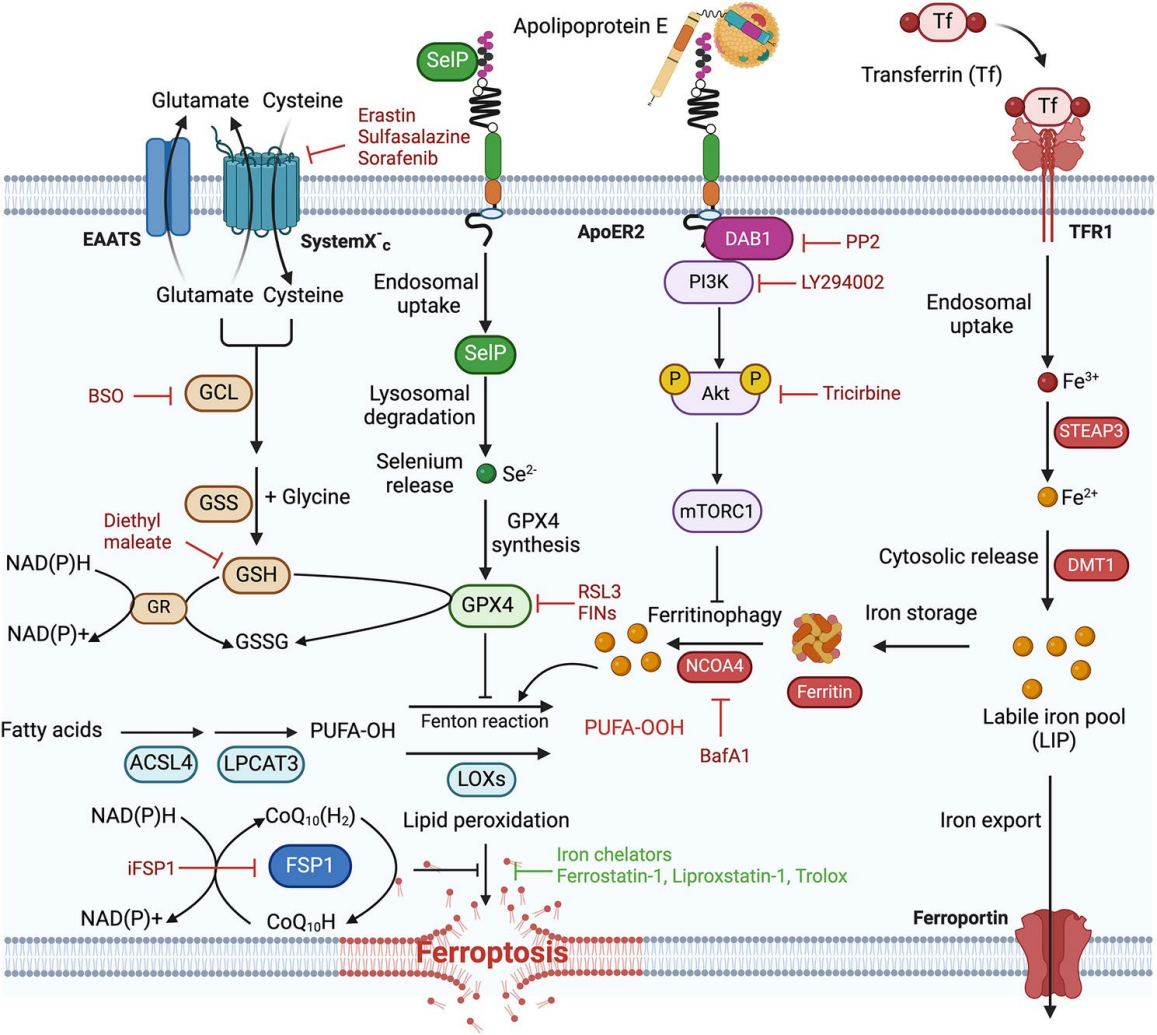
Mechanism of ferroptosis. Schematic description of the ferroptosis pathway highlighting the defense mechanisms mediated by the selenium-dependent protein glutathione peroxidase 4 (GPX4) requiring glutathione (GSH) for activity and ferroptosis suppressor protein 1 (FSP1) requiring NADPH for activity. Iron is required for ferroptosis and can be delivered via transferrin receptor 1 (TfR1) uptake or via autophagic degradation of the iron storage protein ferritin. ApoER2 is linked with selenium uptake and regulation of autophagy. Ferroptosis can be induced by various small molecule inhibitors (highlighted in red) and can be inhibited by radical trapping agents and iron chelators (highlighted in green)

The discovery of ferroptosis as a mechanism of cell death induced by iron-dependent lipid peroxidation provided a mechanism of neurodegeneration linking lipid and iron metabolism, and has been implicated in several neurodegenerative diseases including ischemic stroke, Alzheimer’s and Parkinson’s disease [86]. Ferroptosis is a form of regulated iron-dependent cell death that results from the excessive accumulation of lethal lipid peroxides with polyunsaturated fatty acids (PUFAs) such as arachidonic, linoleic and docosahexaenoic acids as primary substrates [87–89]. Glutathione peroxidase 4 (GPX4) is the primary defense mechanism of the cell against ferroptosis by catalyzing the conversion of lipid peroxides to lipid alcohol in a reaction that consumes glutathione (GSH), but ferroptosis can also be prevented by iron chelators such as deferoxamine and deferiprone, or lipophilic radical-trapping antioxidants, such as ferrostatin-1 and liproxstatin-1(Fig. 4) [87, 90].

ApoE plays an essential role in protecting neurons from lipid peroxidation by shuttling oxidized fatty acids that accumulate during neuronal activity from neurons to astrocytes for storage in lipid droplets or use in energy via β-oxidation [51]. This metabolic association between astrocytes and neurons was shown to be disrupted by the apoE4 isoform [52, 53]. ApoE was shown to directly inhibit ferroptosis and boost GPX4 levels in vitro and in vivo in melanoma [91]. A direct link between apoE and ferroptosis was further discovered in a recent study that identified apoE as the strongest inhibitor of ferroptosis discovered to date, acting within physiological concentrations for the CSF [67]. Under ferroptotic conditions, apoE was shown to prevent iron release from ferritin (that fuels ferroptosis) by activation of the PI3K/AKT pathway that then inhibits the autophagic degradation of ferritin in a process termed ferritinophagy [67]. Another line of evidence linking apoE with ferroptosis and AD was provided by its receptor apoER2, which is required for selenium uptake and GPX4 activity. Low levels of apoER2 were reported as a result of familial AD mutation, leading to increased ferroptosis susceptibility (further described in Sect. 4) [92]. Further evidence linking apoE genotype with ferroptosis was provided in clinical studies that showed elevated lipid peroxides in postmortem brains of *APOE* ε4 carriers [93, 94]. These data collectively support ferroptosis as a possible contributory mechanism of neurodegeneration in AD that could be targeted using small molecule ferroptosis inhibitors.

## Pathologies associated with Apolipoprotein E

ApoE is involved in several pathological mechanisms that contribute to multiple diseases through its influence on multiple biological processes including but not limited to amyloid aggregation, tau pathology, BBB integrity, innate immunity and neuroinflammation. The next section discusses pathologies that are closely related or known to impact on the neuropathology of AD.

### Atherosclerosis and vascular contributions to dementia

The association between the *APOE* ε4 allele and increased risk of cardiovascular diseases and atherosclerosis is well established in the literature and could be partly explained by high circulating levels of cholesterol characteristic of ε4 carriers [95–98]. Indeed, a recent large cohort study confirmed that ε4 carriers were at the highest risk for developing cardiovascular disease and had significantly greater odds of having subclinical atherosclerosis compared with ε4 non-carriers, which was mainly explained by their higher levels of low-density lipoprotein (LDL)-cholesterol [99]. Conversely, the *APOE* ε2 allele is associated with a lower risk of cardiovascular disease and subclinical atherosclerosis, although this protective effect appears largely independent of LDL-cholesterol levels [99].

Atherosclerosis is closely linked to vascular contributions to dementia, as compromised vascular integrity and function due to lipid deposition and inflammation in cerebral blood vessels can exacerbate neuronal damage and cognitive decline. Low apoE levels observed in individuals with atherosclerosis further suggest a potential mechanistic connection [100, 101]. ApoE-deficient mice, widely recognized animal model of atherosclerosis, show pronounced vascular lipid deposition and inflammation, conditions known to contribute to vascular dementia and cognitive impairment, especially under high cholesterol dietary conditions [102–105]. Importantly, macrophages, which express and upregulate apoE during atherosclerosis, play critical roles in regulating vascular inflammation and lipid homeostasis [106]. Bone marrow transplantation studies in mice further demonstrate the protective role of macrophage-derived apoE against atherosclerosis, highlighting the intersection between systemic lipid metabolism, vascular health, and dementia risk [107–109]. Thus, *APOE* allele-dependent cardiovascular effects, particularly through atherosclerosis, may significantly influence vascular pathways contributing to dementia.

### Blood-brain-barrier integrity

The blood-brain barrier (BBB) is essential for maintaining CNS homeostasis by regulating the entry of substances from the blood into the brain. The neurovascular unit of the BBB is composed of a layer of endothelial cells connected by tight junctions, pericytes and astrocytes end feet [110]. Ageing, as well as several neurodegenerative diseases including AD, are associated with reduced BBB integrity, leading to increased permeability and leakage [111, 112].

ApoE deficiency exaggerates age-dependent BBB leakage [113, 114]. Cerebrovascular abnormalities are routinely observed in AD and the ε4 allele is known to increase the risk of cerebral amyloid angiopathy (CAA), which can cause microbleeds and ischemia [115–118]. However, apoE4 removal from astrocytes in an amyloid mouse model of AD conferred benefits by increasing cerebrovascular integrity and blood vessel function, despite increasing CAA, so demonstrating a direct effect of apoE on BBB integrity [119]. Indeed, studies using humanized *APOE knock-in* mice showed that only apoE4-expressing mice (rather than *knock-ins* of the other main isoforms) express a leaky BBB, increased MMP9, impaired tight junctions, and reduced astrocyte end-foot coverage of blood vessels; while removal of astrocyte-produced apoE4 improved all the associated phenotypes [120]. Further studies using humanized *APOE Knock-in* mice showed that BBB breakdown is mediated by apoE4 through activation of a proinflammatory cyclophilin A-matrix metalloproteinase-9 pathway in pericytes that leads to neuronal uptake of multiple blood-derived neurotoxic proteins [121]. Importantly, the same study showed that vascular defects mediated by apoE precede neuronal dysfunction and neurodegeneration [121].

A recent clinical study demonstrated that cognitively unimpaired individuals carrying the *APOE* ε4 allele exhibited early BBB breakdown in the hippocampus and medial temporal lobe compared to non-carriers, with even greater BBB permeability observed in *APOE* ε4 carriers who showed signs of cognitive impairment [122]. Using a BBB pericyte injury biomarker, the study reported that BBB injury predicted future cognitive decline in ε4 carriers but not in non-carriers and correlated with increasing activity of the cyclophilin A-matrix metalloproteinase-9 pathway [122]. Collectively, these results demonstrate a role of apoE in preserving BBB integrity in pericytes and either loss of apoE or expression of the apoE4 isoform induces BBB breakdown that precedes cognitive decline in AD.

### Neuroinflammation

Neuroinflammation is the inflammatory response of immune cells of the CNS to a toxic stimulus that can be protein aggregates such as Aβ or tau, oxidized lipids, stroke, neuronal injury or infection [123]. The neuroinflammatory response involves production of pro-inflammatory cytokines that regulate multiple signaling pathways to promote protection and repair. Yet chronic neuroinflammation may be detrimental and is associated with multiple neurodegenerative diseases including AD [124, 125]. Acute phase response proteins are a cassette of proteins downstream of cytokines that are similarly secreted and can be used to measure the inflammatory state [126]. Acute phase response proteins in CSF increase in MCI and AD subjects. While APOE ε4 carriers do not differ in levels of CSF acute phase response proteins compared to non-carriers, an elevation of these proteins confers greater risk for disease in non- ε4 carriers compared to ε4 carriers, potentially consistent with the threshold for inflammatory damage being lower in ε4 carriers compared to non-carriers [81].

Astrocytes and microglia are the two major immune cells of the CNS with microglia being the brain-resident macrophages of the CNS and the first line of defense against noxious stimuli [125]. Both cells undergo profound changes during inflammation that impact on cell morphology, expression profile and cytokine release, which can be globally classified as pro-inflammatory (A1, M1) or anti-inflammatory (A2, M2) [127]. An additional classification of microglia to disease-associated-microglia (DAM) has been adopted based on the expression of specific markers associated with AD pathology in mouse models and human studies [128–130]. Although apoE expression is mainly restricted to astrocytes, microglial activation induces apoE expression to levels similar to that of astrocytes [131]. In human iPSC-derived microglia, isogenic conversion of *APOE* ε3 to *APOE* ε4 transformed the microglial transcriptome phenotype of normal microglia to DAM [132]. Microglia express multiple receptors that recognize different types of stimuli and mediate activation of Toll-Like Receptors (TLRs) and Triggering Receptor Expressed on Myeloid cells 2 (TREM2), with the latter attracting considerable interest since GWAS studies identified multiple TREM2 variants associated with an increased risk of developing AD [133–136]. ApoE binds TREM2 to facilitate microglia phagocytosis [137–139]. The role of apoE/TREM2 signaling in regulating microglial phagocytosis was confirmed in multiple studies involving AD mouse models and postmortem human brains [140, 141].

An isoform dependent effect of apoE on microglia activation is well reported in animal models of AD and human AD patients [142–145]. However, since apoE isoforms also affect Aβ and tau aggregation, which are known to promote microglial activation, a direct effect of apoE isoforms on microglial activation in AD is complex to conclude [146–148]. Recent studies using specific positron emission tomography (PET) radiotracers for imaging and quantification of microglial activation in living individuals reported increased neuroinflammation in *APOE* ε4 carriers as compared with non-carriers independently of amyloid pathology [142]. Moreover, apoE4 genotype is associated with activated microglia in brain regions associated with early tau deposition [142]. Thus, demonstrating a direct role of apoE and its isoforms in modulating the inflammatory response of microglia in AD.

### Amyloid aggregation

The link between apoE and Aβ production, aggregation and clearance is one of the most widely investigated fields in AD over the last decades. Although the association has been firmly established, the underlying mechanisms are still not clear especially regarding clearance mechanisms [22, 149–152]. Aβ is formed by the sequential cleavage of the transmembrane amyloid precursor protein (APP) via β- and γ-secretase to generate monomeric Aβ, which then polymerizes to form soluble oligomers, protofibrils and insoluble amyloid fibrils, which are the main components of amyloid plaques in AD [153, 154]. Aβ deposition starts in the preclinical stage of the disease and progresses with age with compelling evidence linking apoE isoforms with this process. *APOE* ε4 carriers have increased Aβ plaque deposition and earlier onset of amyloid pathology than non-carriers, while *APOE* ε2 carriers show delayed deposition and less Aβ pathology [155–159]. This apoE effect was reproduced in multiple AD mouse models with an isoform-dependent increase in amyloid aggregation with ε4 > ε3 > ε2 [160–163].

Suppression of apoE expression in mouse models of AD was shown to significantly reduce Aβ plaque deposition, suggesting that apoE actively promotes amyloid aggregation [164]. Interestingly, recent mouse studies indicate that apoE impacts on Aβ plaque initiation since suppression of apoE expression using antisense oligonucleotides (ASOs) in AD transgenic mice reduced plaque load only when delivered prior to plaque onset but had no effect once the plaques were established [165]. In line with those results, apoE4 overexpression at in early age induced a strong increase in Aβ plaque load and Aβ levels in AD transgenic mice, but starting apoE4 overexpression after plaque onset did not influence plaque load and Aβ levels [166]. Thus, apoE appears to promote Aβ aggregation during the early seeding stage.

But how does apoE promote amyloid seeding? One possible mechanism may involve direct binding of Aβ to apoE in a process impacted by apoE lipidation. Decreasing lipidation of apoE decreases binding to Aβ and abolishes the isoform difference in Aβ binding, while clearing non-lipidated apoE using specific antibodies suppresses amyloid aggregation in AD mouse models [167, 168]. Conversely, increasing apoE lipidation via ABCA1 overexpression in AD mice reduced Aβ plaque load with the opposing effect in ABCA1-deficient mice [169, 170].

Recently, the mechanism of protection conferred by another variant of apoE: apoE3 Jacksonville variant, was attributed to enhanced apoE lipidation, leading to decreased amyloid deposition and reduced plaque load [171]. Interestingly, introducing the Jacksonville amino acid change (V236E in lipid binding region, Fig. 1) in the apoE4 protein not only reduced Aβ aggregation but also reduced apoE4 self-aggregation [171]. This finding suggests that apoE oligomerization may serve as a ground structure that binds and facilitates Aβ aggregation, with non-lipidated apoE more prone to oligomerization than lipidated apoE. Indeed, biochemical characterization of recombinant apoE with or without lipid complex showed that apoE has the tendency to self-aggregate in solution and this process is inhibited by lipidation [49]. ApoE lipidation increases Aβ binding by 5- to 10-fold and native apoE3 exhibits 2-3-fold higher affinity for Aβ than apoE4 [168].

A recent study using single molecule imaging confirmed that all apoE isoforms associate with Aβ to form fibrils and that the presence of non-lipidated apoE slowed the aggregation process and formed large early-stage Aβ co-aggregates, supporting a role of non-lipidated apoE in stabilizing intermediate complexes with Aβ [50]. This finding was supported by the detection of poorly lipidated apoE4-Aβ co-aggregates in AD brains [50]. The study also performed uptake assays using human induced pluripotent stem cells (hiPSC)-derived microglia and showed that colocalization of apoE with Aβ aggregates promotes Aβ uptake and clearance, while clearance of non-lipidated apoE-Aβ complexes was slow and may lead to greater deposition and inflammatory response [50]. In summary, there is strong evidence for apoE mediating Aβ clearance through complex formation, with apoE4 or poorly lipidated apoE being cleared inefficiently by innate immune cells.

Additional mechanisms to explain the impact of apoE on amyloid pathology include an apoE isoform-dependent effect on Aβ overproduction from APP and impact on the activity of enzymes involved in Aβ degradation [172, 173].

### Tauopathy

Tau pathology is another major hallmark of AD that is defined by the accumulation of hyperphosphorylated tau protein in neurons, which progress to form intracellular aggregates termed neurofibrillary tangles (NFTs). Tau imaging and postmortem analyses revealed a strong spatial and temporal correlation between tau pathology and brain atrophy in AD, which tightly associates tau phosphorylation with the neurodegeneration process in AD [174–176]. Multiple studies report that the apoE4 isoform is linked to an increase in phosphorylated tau (p-tau) and tau-associated neurodegeneration in AD [177–179]. But the effect of apoE4 on tau-mediated neurodegeneration is also linked to brain atrophy in other tauopathies such as frontotemporal dementia (FTD) and chronic traumatic encephalopathy (CTE) [180, 181]. Thus, demonstrating that apoE4 is associated with tau pathology both in the presence and absence of Aβ [178, 182].

ApoE4 association with tau was further illustrated in studies using transgenic mouse models of tauopathies [146, 183]. Specifically, crossbreeding between humanized *APOE Knock-in* mice and the P301S tau mouse model of tauopathy (PS19 mouse overexpressing the P301S tau mutant) showed that only apoE4 expression exacerbated tau pathology and associated neurodegeneration when compared to the other isoforms [146]. Interestingly, deletion of the endogenous *apoE* gene in the PS19 mouse model rescued neurodegeneration without affecting p-tau and total tau levels, indicating that apoE may affect other pathological processes such as neuroinflammatory response by microglia [146, 184]. Collectively, these results reveal a role of apoE4 in accelerating tau spreading and tau-associated neurodegeneration independently of amyloid but this does not exclude an additional impact of Aβ on tau pathology and both effects are difficult to dissect. The mechanisms involved are still under debate, but recent data gained from familial AD cases and replicated in mouse studies provide important clues on the involvement of apoE receptors in tau spreading, neuroinflammation and associated neurodegeneration, discussed next.

## Intersection with familial Alzheimer’s disease

Most of the knowledge gained on amyloid pathology originated from case studies of familial AD, which are autosomal dominant phenocopies of sporadic AD caused by mutations in genes directly related to amyloid production: amyloid precursor protein (*APP*), Presenilin1 (*PSEN1*), and Presenilin 2 (*PSEN2*). Familial AD patients share the same pathological hallmarks of sporadic late-onset AD including high amyloid plaque burden and tau pathology but disease onset is usually before 50 years of age.

ApoE pre-dominantly drives risk for late-onset AD since the ε4 carriers are highly represented in late-onset AD cases [15]. Evidence for the involvement of apoE in familial AD was observed in early studies of Down syndrome cases, in which the *APP* gene is triplicated, where the presence of the *APOE* ε2 genotype was associated with reduced risk of AD and delayed onset of dementia [185–187]. However, the most direct evidence was obtained from studies on a large Columbian family that included the largest known cohort of FAD patients (more than 1000) due to a single mutation in the *PSEN1* gene (*PSEN1 E280A*) [188]. *PSEN1 E280A* carriers from this cohort present mild cognitive impairment (MCI) at a median age of 44 and dementia at 49 years of age [188]. Earlier studies on this cohort showed that the *APOE* ε4 allele was associated with accelerated cognitive decline and earlier onset of dementia compared to non-carriers [189]. This *APOE* ε4 effect was confirmed in a recent study from the same cohort that included a larger sample size (675 mutation carriers and 594 controls), and further reported that the *APOE* ε2 genotype was associated with a delay in age-dependent cognitive decline as compared to non-carriers [190]. Recent data from the same cohort identified further evidence linking apoE and apoE receptors to an unprecedented robust protection from familial AD (covered in the next sections).

### Apolipoprotein E Christchurch variant

Recently, a rare variant known as apoE3 Christchurch (R136S) has been identified, offering significant protection against familial AD [32]. This variant was first associated with a lipid disorder named type III hyperlipoproteinemia in humans [191]. In 2019, a homozygous *APOE* ε3 *Christchurch* allele was discovered in a Colombian woman carrying the *PSEN1 E280A* mutation, who instead of developing cognitive impairment at 44 years of age, presented with mild cognitive impairment in her late 70s and thus was protected from developing dementia for nearly 3 decades [32]. Remarkably, imaging data and postmortem analyses showed very limited tau pathology, low hippocampal atrophy and normal glucose metabolism despite extremely high levels of amyloid plaques, suggesting that apoE Christchurch may directly prevent tau pathology spreading even in the presence of a high amyloid load [32, 192].

Although, it was a single case study, the robust protection conferred by the apoE Christchurch variant was reproduced in different laboratories using mouse models of tauopathy and amyloid pathology. Accordingly, introducing the Christchurch mutation in apoE4 was sufficient to prevent tau pathology and associated tau-neurodegeneration in PS19 mice in the absence of amyloid pathology [193]. In another series of experiments, apoE3 Christchurch was effective in preventing tau pathology and associated neurodegeneration when human fibrillar tau was injected in an amyloid mouse model of AD [194]. A common feature of all studies including the human case report was a decrease in neuroinflammation markers and microglia activation, suggesting a role of the Christchurch variant in modulating the neuroinflammatory response of the brain [192–194]. Additional clinical studies from the Colombian cohort further revealed that individuals with one copy of the *APOE* ε3 *Christchurch* variant experienced a median delay of five years in disease onset compared to non-carriers [195]. This is consistent with *APOE* ε3 *Christchurch* variant exerting a competitive protective role even in the presence of endogenous apoE with greater protection against AD being dose-dependent [196].

### Link to Apolipoprotein E receptors

The world second case of complete protection from familial AD was also discovered in the Columbian familial AD cohort and was identified in a male patient carrying the *PSEN1 E280A* mutation who was first diagnosed with MCI at the age of 67 years and progressed to mild dementia by the age of 72 years [197]. Genetic analysis identified a heterozygous mutation in the ligand of apoER2, reelin, which was named *REELIN COLBOS* (H3447R) [197]. Neuroimaging analyses revealed high amyloid plaque burden as compared to younger cognitively impaired *PSEN1* mutation carriers from the same cohort, reminiscent of the previously reported apoE Christchurch case [197]. Tau tangle burden was also reduced in many brain regions typically affected in cognitively impaired mutation carriers, but tau pathology in the inferior temporal lobe and brain atrophy were at similar levels between the patient and typical cognitively impaired mutation carriers from the cohort, and severe amyloid and tau pathology was later confirmed at postmortem [197].

COLBOS and Christchurch mutations both protected against cognitive decline in FAD patients despite an unusually heavy amyloid plaque burden [197]. One major difference between the expression of these mutations was that the *COLBOS* carrier had cognitive preservation despite heavy tau pathology and brain atrophy, suggesting additional mechanisms that protected against tau-mediated neurodegeneration. The COLBOS mutation was linked to stronger activation of the canonical reelin target Dab1, and this signaling pathway was shown to directly lead to reduction in tau phosphorylation and amelioration of the phenotype in a mouse model of tauopathy [197]. Studies of the apoE Christchurch variant indicated that its protective effect might be mediated by its reduced binding affinity to heparan sulfate proteoglycans, which decreases it binding to LRP1 and so inhibits spread of tau pathology [193, 194]. However, the COLBOS study suggest that different apoE receptors could be involved in both spreading of tau pathology and protection from tau-mediated neurodegeneration. A recent genetic study on the Colombian cohort identified LRP1B, as an additional member of the LDL receptor family that may impact on age at onset of AD [198]. These findings underscore the benefits of investigating genetic associations with the rare form of familial AD and their potential in discovering new pathways that inform disease mechanism.

## Apolipoprotein E risk across sex and population ancestry

Emerging evidence across multiple large-scale studies reveals that the impact of the *APOE* genotype on AD risk is far more nuanced than previously understood, shaped by an intricate interplay between ancestry, sex, and epigenetic context. While the *APOE* ε4 allele is a well-established genetic risk factor for AD, its penetrance varies significantly across populations. For example, studies consistently show that East Asians carrying *APOE* ε4 exhibit the highest AD risk, followed by non-Hispanic Whites, whereas individuals of African ancestry demonstrate attenuated risk, even when *APOE* ε4 allele frequency is high [199–201]. This disparity is partly explained by local genetic ancestry and regulatory variation: African local ancestry (ALA) at the *APOE* locus is associated with reduced apoE4 expression, especially in astrocytes, likely mitigating its neurotoxic effects [201, 202]. Conversely, European local ancestry (ELA) is linked to increased apoE4 expression and greater chromatin accessibility at the *APOE* promoter, potentially exacerbating AD risk [202].

Further, ethnic substructure within admixed populations, such as Hispanics, introduces additional complexity. Risk associated with *APOE* ε4 varies substantially by regional ancestry, with South American groups showing strong associations, while Mexican cohorts exhibit negligible *APOE* ε4-related AD risk [203]. These findings reinforce the need for population-specific modeling in both genetic risk prediction and therapeutic targeting. Moreover, sex adds another critical layer: while *APOE* ε2 is generally considered protective, recent data indicate that this effect is sex-specific and ancestry-modulated—strong in non-Hispanic White men but not observed in women or in Black individuals [204].

Together, these findings challenge a one-size-fits-all approach to apoE-targeted therapies and underscore the urgent need to consider ethnicity-specific approaches when evaluating apoE-related risk profiles and developing tailored therapeutic strategies for AD.

## Therapeutic strategies targeting Apolipoprotein E

Given the major impact of the *APOE* ε4 allele on AD risk and the accelerated cognitive decline and spread of amyloid and tau pathologies associated with this allele, there is a consensus in a large research consortium group that lowering the apoE4 protein should be a therapeutic target for the treatment of AD [205]. Recent data support also expression of apoE2 as a therapeutic target in AD [15]. Thus, lowering apoE4 expression or inducing the expression of apoE2 and the recently discovered apoE Christchurch variant present as valid therapeutic targets in AD. The strategies used to achieve those targets along with other therapeutical targets are summarized in the next sections.

### Lipidation

Because of the poor lipidation of apoE4 compared to other apoE isoforms, initial apoE-based therapeutic strategies consisted of increasing apoE lipidation by activating multiple signaling pathways targeting liver X receptor (LXR), retinoid X receptor (RXR) and peroxisome proliferator-activated receptor (PPAR), which control the expression of multiple genes involved in lipid biosynthesis such as ABCA1 and ABCG1 [206, 207]. One of the widely tested drugs is Bexarotene, which is an RXR/LXR agonist that initially showed promising results in animal models of AD, increasing amyloid clearance and improving behavioral deficits [208, 209]. However, follow-up studies showed contradicting results, with no benefits in AD mice [210–212]. Two randomized, placebo-controlled clinical trials, which explored the effect of bexarotene in healthy people and AD patients showed also no clinical benefits [213, 214]. Other compounds tested include the RXR agonists: LG100268, T0901317, GW3965 or ABCA1 agonists such as CS-6253, which showed benefits in cell and animal models of AD [215–218]. Notably, current strategies targeting lipids are not brain-specific and affect peripheral lipid biosynthesis, which is associated with increased triglyceride levels and side effects such as liver steatosis [219]. Future strategies specifically targeting brain lipid biosynthesis genes may prevent off-target effects caused by changes in peripheral lipids.

### Structure correction

The amino acid change from cysteine to arginine in apoE4 results in domain-domain interaction within the protein involving the Arg61 and Glu255 residues, which induces a conformational change that may explain the poor lipidation associated with this isoform in comparison to apoE3. Evidence supporting a role of domain interaction in mediating apoE4 biology was obtained from in vitro studies in neuronal N2A cells expressing R61T mutated apoE4, which abolishes the domain interaction. R61T mutant apoE4 did not induce mitochondrial dysfunction in contrast to apoE4 expression in the same cells [220]. Thus, small molecules that disrupt domain-domain interaction in apoE4 have been proposed as a therapeutic strategy to structurally modify the apoE4 protein to an apoE3-like structure [221].

Several small molecule structural correctors such as GIND25 and PH002, which are phthalazinone derivatives that directly bind to apoE4, rescued impaired apoE4 mobility and protein trafficking that occur when apoE4 is expressed in neurons [222]. In particular, PH002 rescued apoE4-induced impairment of dendritic spine development in primary neurons [222]. Further studies using human iPSC-derived neurons showed that PH002 decreased production and secretion of both p-tau, Aβ_40_ and Aβ_42_ only when apoE4 was expressed, demonstrating that the beneficial effect is mediated by apoE4 structural changes [223]. These results suggest that structural modification of apoE4 could be beneficial in preventing some biological effects of apoE4 in neurons. Data on the effects of apoE4 structure correctors in vivo are still lacking.

### Modulation of expression levels

Decreasing apoE expression was suggested as a therapy since *APOE* deletion reduces Aβ deposition in amyloid mouse models and rescues neurodegeneration in tauopathy mouse models [146, 164, 224]. Several strategies were tested in AD animal models, including use of antisense oligonucleotides (ASO) to target apoE mRNA or using apoE antibodies to target the apoE protein. Intracerebroventricular administration of ASOs targeting apoE to APP/PS1-21 humanized *APOE* ε4 homozygote mice was effective in lowering adult Aβ plaque burden when administered at birth but did not affect plaque burden when administered at the onset of plaque deposition (6 weeks of age) [165]. Repetitive injection of apoE-antibodies was shown to decrease Aβ plaque deposition and insoluble Aβ accumulation in the cortex and hippocampus in an amyloid mouse model of AD [225]. A more specific immunotherapy approach that used the HAE-4 antibody, which preferentially binds the non-lipidated form of apoE was shown to reduce Aβ plaque deposition in APP/PS1-21/*APOE* ε4 mice when administered via intracerebroventricular injection [167]. Collectively, the evidence supports suppressing apoE in *APOE-*ε4 carriers as a therapeutic strategy, but further experiments are required to verify efficacy and off-target effects of ASOs and immunotherapies targeting apoE.

### Targeting ApoE receptors

ApoE binding to apoE receptors can mediate endocytosis and clearance of amyloid and tau by LDLR and LRP1 receptors. Deletion of either receptor increases amyloid deposition but apoE binding to other receptors such as VLDLR and apoER2 could serve to activate protective signaling, as shown with ligands such as reelin-COLBOS that protects against AD pathology by over-activating the Dab1 signaling pathway [197, 226, 227]. ApoE4 was shown to disrupt the reelin signaling pathway by reducing apoER2 levels at the plasma membrane [228]. The structural properties of apoE4 caused delayed dissociation from apoE receptors, impairing endolysosomal trafficking, and leading to decreased recycling of apoER2 to the plasma membrane [228]. Interestingly, acidification of endosomes, by specific inhibitors or by genetic deletion of the endosome-specific sodium-hydrogen exchanger 6 (NHE6), not only restored plasma membrane apoER2 levels but also suppressed amyloid deposition even in the absence of apoE4 [228]. Therefore, apoE4-induced impaired endolysosomal trafficking may hamper protective signaling and reduce amyloid clearance via different apoE receptors.

Brain apoER2 levels are decreased in familial and sporadic AD cases, and low apoER2 levels increases the vulnerability of neurons to ferroptotic death [92]. Furthermore, LDLR overexpression in the brain suppressed amyloid and tau burden in AD mouse models [26, 229]. Another approach to target apoE receptors is the use of apoE peptides that include motifs corresponding to the apoE receptor binding region. Several apoE-mimetic peptides reduced amyloid plaque burden and improved cognition in multiple AD mouse models [230–232]. Thus, targeting apoE receptors and in particular apoER2 signaling could be explored as a new avenue in the development of apoE-based therapies for AD.

### Gene therapies

Adeno-associated virus (AAV) mediated gene delivery of apoE is one of the most promising gene therapies pursued by many laboratories. Specifically, AAV-mediated overexpression of the apoE2 variant in the brain reduced amyloid deposition, synaptic loss and microglial overactivation in AD mouse models [233]. More importantly, the beneficial effect of apoE2 expression in the brain was achieved in the presence of endogenous expression of the apoE4 risk variant, indicating that apoE2 can counteract some of the detrimental effects of apoE4 in vivo [233]. Based on those results, a phase I clinical trial exploring AAV-mediated expression of apoE2 via intracisternal injection was explored in *APOE* ε4 homozygous with promising initial results from a low number of enrolled participants reported in press but until now only safety data primates were published [234]. Conversely, as an attempt to worsen pathology by apoE4 overexpression, AAV-mediated expression of apoE4 did not alter tau burden in tauopathy mouse models [235].

A major advancement in the development of apoE-based gene therapies was recently reported with the AAV-mediated expression of an apoE variant combining the apoE2 and Christchurch variant in amyloid and tau-based AD mouse models. Accordingly, AAV-mediated delivery of apoE2 in CNS was found to decrease amyloid load in APP/PS1/*APOE* ε4 mice but was not effective in preventing tau pathology in PS19/*APOE* ε4 mice [236]. In contrast, AAV-mediated apoE2 Christchurch delivery in CNS was effective in inhibiting both amyloid and tau pathology as well as neurodegeneration in AD mouse models [236]. The discovery of apoE Christchurch and the beneficial effect of the hybrid apoE2 + Christchurch variant in AD mouse models represent major breakthroughs in the field and could accelerate the development of clinical trials to assess the efficacy of AAV-mediated apoE therapy in AD [236].

### Other strategies

Additional apoE-based strategies include disruption of Aβ interaction with apoE by using Aβ-mimetic peptides that have strong affinity for apoE. Such peptides were shown to reduce amyloid deposition and tau accumulation in AD mouse models [237]. Although apoE-based therapies are targeting the CNS, it should be noted that several studies reported contributions of peripheral apoE to AD pathology. Indeed, restoration of apoE expression only to the periphery improves cognition and restores synaptic defects in *APOE KO* mice, suggesting that plasma apoE/lipid levels can influence cognition and synaptic function independent of apoE expression in the CNS [238]. Expression of apoE4 in the periphery was also shown to enhance AD pathology and impair cognition, likely by compromising cerebrovascular functions [239]. The same study showed that apoE3 plasma from young mice improved cognition and reduced age-related BBB damage when transfused into aged mice [239]. Contribution of peripheral apoE was also confirmed by a human cohort study showing that high apoE levels in HDL that lacked apoC3, which is an apolipoprotein secreted by liver and small intestine, was associated with better cognitive function and lower dementia risk [240]. Knowing that plasma apoE levels are decreased in *APOE* ε4 carriers and increased in *APOE* ε2 carriers, future studies could explore the benefit of therapeutic strategies targeting plasma apoE levels in AD.

## Concluding remarks and future perspectives

Current therapeutic strategies targeting amyloid plaque removal have led to the FDA-approval of three antibody-based drugs that achieved a robust reduction in amyloid plaque load in the brain but the benefits on cognitive decline are modest and there are concerns about their safety [241, 242]. Anti-amyloid immunotherapies increase the incidence of amyloid-related imaging abnormalities (ARIA), that may be asymptomatic or lead to serious secondary effects [243]. *APOE* ε4 carriers are more susceptible to ARIA than non-carriers, thus limiting the treatment of this group of patients, who are most vulnerable to AD. Therefore, there is an urgent need to find alternative treatment approaches for AD. The biology of apoE provides numerous such potential treatment targets.

ApoE stands at the intersection of numerous pathological processes central to AD, including amyloid and tau aggregation, neuroinflammation, blood-brain barrier integrity, and vascular health. Across all these domains, the influence of apoE is remarkably isoform-specific, with the apoE4 variant consistently associated with increased pathology and heightened risk. Large-scale genomic and transcriptomic studies show that *APOE* ε4 risk allele is attenuated in individuals of African ancestry, likely due to regulatory differences that limit apoE expression in key cell types such as astrocytes. This highlights the importance of ancestry-informed cohort studies and the need for inclusive research that reflects the diversity of AD patients worldwide.

**Fig. 5:**
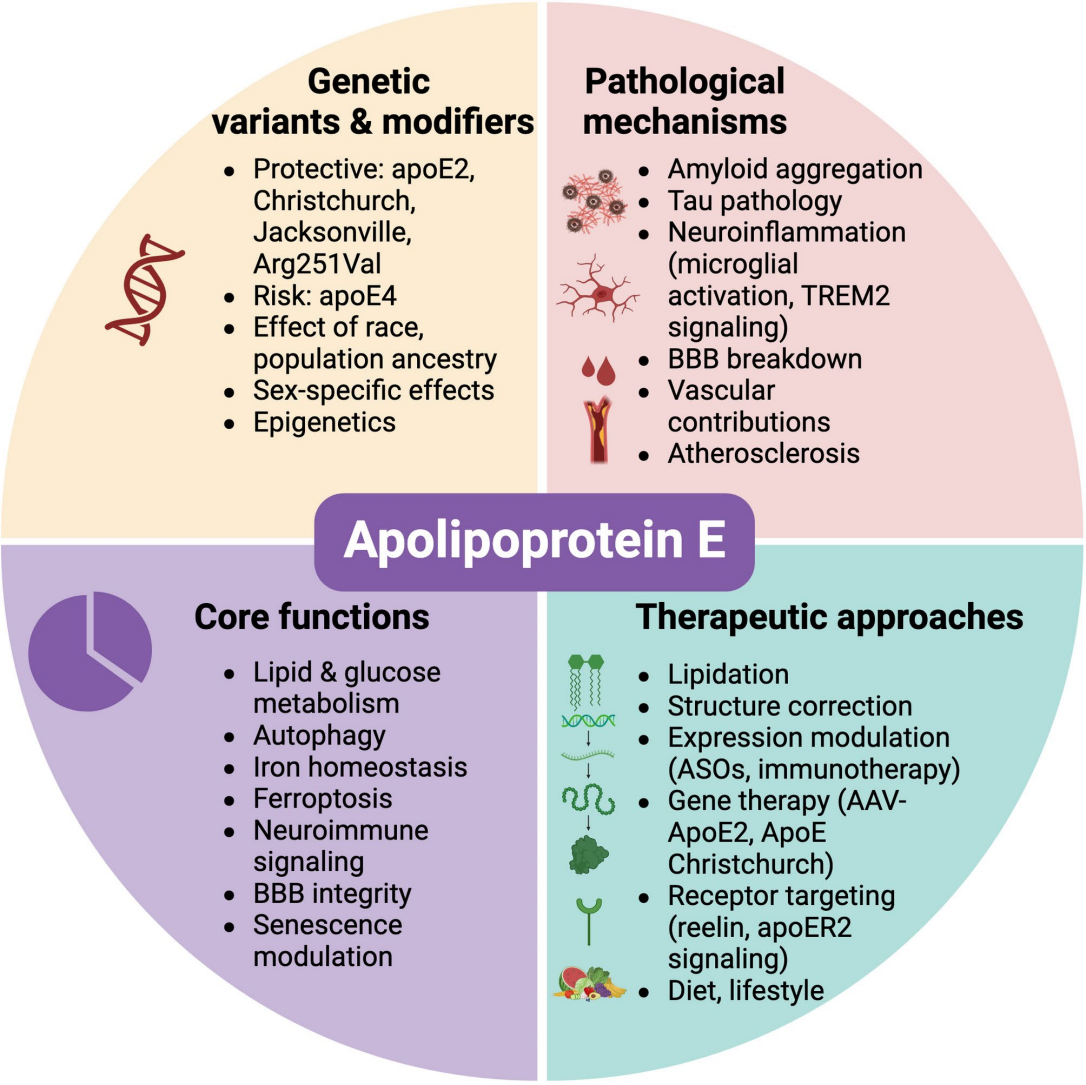
ApoE biology, intersection with disease and therapeutic approaches

Importantly, findings from both sporadic and familial AD cases—including the extraordinary protection observed in carriers of the Christchurch and COLBOS variants—underscore the therapeutic potential of targeting apoE structure, function, and receptor interactions. These cases exemplify how modifying a single residue or signaling pathway can alter the disease trajectory, even in individuals with high amyloid burden (Fig. 5).

Given the extensive and multidisciplinary nature of apoE research, this review has focused primarily on the isoform-dependent roles of apoE in AD pathogenesis and therapeutic strategies. However, several important aspects of apoE biology, including the influence of lifestyle factors, vascular health, and systemic metabolic regulation, are not covered in detail here but are critical for a comprehensive understanding. Topics related to how diet, physical activity, and other modifiable risk factors interact with *APOE* genotype are covered in reviews such as Mahley & Rall (2021) and Kivipelto et al. (2018), which discuss apoE’s responsiveness to environmental modulation [244, 245].

In conclusion, targeting apoE remains one of the most promising avenues in the pursuit of Alzheimer’s disease therapeutics. To fully realize its potential, we must deepen our understanding of apoE’s interactions with its receptors and the downstream signaling pathways that influence both neuronal and glial function. A particularly compelling approach involves leveraging rare, naturally protective variants of apoE—such as apoE2 and apoE Christchurch—as templates for therapeutic development. These variants offer unique insights into protective mechanisms that counteract neurodegeneration, providing valuable clues to replicate their effects pharmacologically. Additionally, modulating receptor signaling pathways to preferentially activate protective responses holds tremendous promise. By integrating these strategies, we can move toward a new generation of interventions that not only slow disease progression but may also mitigate the impact of Alzheimer’s disease across diverse genetic backgrounds, heralding a future with more effective, targeted treatments.

## Data Availability

Not applicable.

## Abbreviations

APOE: Apolipoprotein E
AD: Alzheimer’s disease
FAD: Familial Alzheimer’s disease
MCI: Mild cognitive impairment
APP: Amyloid precursor protein
PSEN1: Presenilin 1
PSEN2: Presenilin 2
Aβ: Amyloid beta
NFT: Neurofibrillary tangles
ABC: ATP-binding cassette
ABCA1: ATP-binding cassette transporter 1
ABCG1: ATP-binding cassette subfamily G member 1
LDLR: Low-density lipoprotein receptor
VLDLR: Very low-density lipoprotein receptor
LRP1: Low-density lipoprotein receptor-related protein 1
APOER2: Apolipoprotein E receptor 2
HSPGs: Heparan sulfate proteoglycans
TREM2: Triggering receptor expressed on myeloid cells 2
CNS: Central nervous system
BBB: Blood-brain-barrier
iPSC: Induced pluripotent stem cell
DAM: Disease associated microglia
AAV: Adeno-associated virus
